# The prognostic impact of peripheral blood eosinophil counts in metastatic renal cell carcinoma patients treated with nivolumab

**DOI:** 10.1007/s10238-024-01370-8

**Published:** 2024-05-23

**Authors:** Akihiro Yoshimura, Akira Nagahara, Yu Ishizuya, Yoshiyuki Yamamoto, Koji Hatano, Atsunari Kawashima, Yasutomo Nakai, Masashi Nakayama, Kazuo Nishimura, Norio Nonomura, Taigo Kato

**Affiliations:** 1 Departments of Urology, Osaka University Graduate School of Medicine, 2-2 Yamadaoka, Suita, Osaka 565-0871 Japan; 2 Department of Urology, Osaka International Cancer Institute, Osaka, Japan

**Keywords:** Renal cell carcinoma, Immune checkpoint inhibitor, Eosinophil, Immune-related adverse event

## Abstract

Although immune checkpoint inhibitors (ICIs) have gained approval for metastatic renal cell carcinoma (mRCC), the response rate is still limited. Therefore, it is urgent to explore novel markers of responses to ICIs that can help assess clinical benefits. Recently, it has been noted that peripheral blood eosinophil counts are an independent factor correlated with clinical outcome of ICIs in some types of cancer. We investigated peripheral blood absolute eosinophil counts (AECs) at baseline and 4 weeks after the initiation of nivolumab for mRCC patients between February 2016 and May 2022. In addition, we examined clinicopathological features including irAEs and analyzed the correlation between AECs and clinical efficacy of nivolumab. The median progression-free survival (PFS) and overall survival (OS) for all patients were 5.7 and 25.5 months, respectively. The median AECs in patients with irAEs were significantly higher at baseline and 4 weeks after the treatment compared to those without irAEs (*p* < 0.001 and *p* = 0.001). With the cutoff value of AECs of 329 cells/µL at 4 weeks after the treatment for prediction of irAEs, high-AECs groups had significantly higher number of responders compared with that in low-AECs group (*p* < 0.001). Accordingly, the PFS and OS were significantly better in patients with high-AECs group than those in low-AECs group (*p* = 0.03 and *p* = 0.009). High-AECs at 4 weeks after the treatment serve as the prominent surrogate marker associated with the incidence of irAEs and better clinical outcome in mRCC patients receiving nivolumab.

**Supplementary Information:**

The online version contains supplementary material available at 10.1007/s10238-024-01370-8.

## Introduction

Renal cell carcinoma (RCC) accounts for about 2–4% of all types of cancer worldwide [1]. The 5-year specific survival in patients with early stage is reported to be 90%, but once metastasized, the 5-year survival plummets to 14%, although 17% of RCC patients present with the evidence of distant metastasis at initial diagnosis [2, 3]. In addition, 20% of RCC patients who undergo surgical resection of localized RCC eventually develop distant metastases, necessitating subsequent therapeutic interventions including immune checkpoint inhibitors (ICIs)-based treatments [4].

ICIs have been widely used in the treatment of many types of cancer and have remarkably improved the prognosis of cancer patients. However, the majority of patients may not benefit from the therapy with ICIs and sometimes experience severe immune-related adverse events (irAE). To date, numerous biomarkers, including PD-L1 expression, tumor mutation burden, and tumor-infiltrating lymphocytes in cancer tissues, have been suggested to play a crucial role in influencing the therapeutic response to ICIs [5]. However, these markers mainly focus on the tumor state at the time of diagnosis and sometimes produce contradictory outcomes. Therefore, there is an urgent need for noninvasive biomarkers that can predict the efficacy of ICIs, aiming to prevent unnecessary treatments.

Various factors have been investigated to predict response, and an association between neutrophil lymphocyte ratio (NLR) and therapeutic response has been reported as a typical marker in peripheral blood in several cancer types including RCC [6–8]. Recently, several studies reported that peripheral eosinophil counts may predict the incidence of irAE and are associated with the response to ICIs in several types of cancer [9–16]. However, there have been not fully understood the association between eosinophil counts and clinical prognosis in RCC, especially with ICIs treatment.

In the present study, we demonstrated that metastatic RCC (mRCC) patients who experienced severe irAE had consistently higher eosinophil counts before and at any time point after the nivolumab when compared to those without. Furthermore, the high absolute eosinophil counts (AECs) at an early time point were significantly associated with a better clinical outcome in mRCC patients. Collectively, these markers may concisely and practically predict clinical response in mRCC patients with anti-PD-1 inhibitor.

## Materials and methods

### Patients

We retrospectively investigated data from 83 mRCC patients treated by nivolumab at 2 institutions (Osaka International Cancer Institute and Osaka University Medical Hospital) from February 2016 to May 2022. For each patient, we collected baseline demographic and clinical data including age, gender, with or without nephrectomy, histological type, Karnofsky Performance Status (KPS), International Metastatic RCC Database Consortium (IMDC) risk classification, metastatic site and treatment line of nivolumab. Peripheral blood AECs were measured at baseline, 2, 4 and 6 weeks after initiation of nivolumab.

Tumor response was evaluated every 8–12 weeks, according to the Response Evaluation Criteria in Solid Tumors version 1.1 (RECIST ver1.1), using computed tomography. Adverse events were evaluated by Common Terminology Criteria for Adverse Events version 5.0 (CTCAE ver5.0). For each patient, the best response during treatment including complete response (CR), partial response (PR), stable disease (SD) or progressive disease (PD) was measured. Progression-free survival (PFS) was defined as the time from the initiation of nivolumab to documented progression or death of any cause. Overall survival (OS) was defined as the time from the start of nivolumab to documented death of any cause or last contact.

The study was approved by the Institutional Review Board of each institution (approval number 018–0003 in Osaka University Hospital and 18,042 in Osaka International Cancer Institute) and was conducted in accordance with the Declaration of Helsinki.

### Treatment

The patients received 240 mg/body of nivolumab every two weeks or 480 mg/body every four weeks until disease progression, clinical deterioration, unacceptable toxicity, or patient’s refusal.

### Statistical analysis

We divide the patients into two groups according to the best response and irAE grades. The AECs of two groups were compared using the *t* test. PFS and OS were estimated by the Kaplan–Meier method and compared with the log-rank test. The prognostic significance of certain parameters was assessed by the Cox proportional hazards regression model. Differences were considered significant at *p* value < 0.05. All statistical analyses were conducted in JMP-software ver. 17.0 (SAS Institute, Cary, NC, USA).

## Results

### Patients’ characteristics

The clinical characteristics of all patients are shown in Table 1. The median age at treatment was 64 years (range, 27–83). Nephrectomy had been performed in 74 patients (89%). The most predominant histological type was clear cell RCC (82.0%). With respect to the IMDC risk classification, 7, 67, and 25% were classified as favorable-, intermediate-, and poor-risk, respectively. The treatment line of nivolumab was 2nd, 3rd and 4th or later in 35, 28 and 20 patients, respectively.

**Table 1:** Baseline characteristics of metastatic renal cell carcinoma patients treated with nivolumab

		*n* = 83
Age(years) at treatment, median (range)		64 (27–83)
Gender	Male Female	56 (67%) 27 (33%)
Prior nephrectomy	Yes No	74 (89%) 9 (11%)
Histological type	Clear cell Papillary Chromophobe Other Unknown	68 (82%) 5 (6%) 2 (2%) 3 (4%) 5 (6%)
KPS	100 90 80 70 50	41 (49%) 29 (35%) 4 (5%) 4 (5%) 5 (6%)
IMDC risk classification	Favorable Intermediate Poor	6 (7%) 56 (68%) 21 (25%)
Metastatic site	Lung Lymph Node Bone Pancreas Adrenal gland Liver Brain	48 (58%) 24 (29%) 26 (31%) 10 (12%) 9 (11%) 9 (11%) 2 (2%)
Treatment line of nivolumab	2nd 3rd 4th or later	35 (42%) 28 (34%) 20 (24%)
Duration of nivolumab treatment (months), median (range)		5.6 (0.7–35.7)
Follow-up period (month), median (range)		19.6 (0.8–59.1)
Objective response	CR PR SD PD	7 (8%) 17 (21%) 30 (36%) 29 (35%)
Time to irAE (day), median (range)		90 (21–595)

### Clinical outcomes of patients with nivolumab

The median duration of nivolumab treatment was 5.6 month (range, 0.7–35.7), and the median follow-up was 19.6 month (range, 0.8–59.1). During the observational period, 39 patients died from cancer. Overall, the median OS was 25.5 months, and the median PFS was 5.7 months (Supplementary Fig. 1). Among 83 patients, CR and PR were achieved in 7 (8.4%) and 17 patients (20.5%), respectively, and SD was observed in 30 patients, resulting in objective response rate (ORR) of 28.9% and disease control rate (DCR) of 65.0%. In the present study, we defined responders as patients with CR, PR.

### Safety analysis

In the present study, 22 patients (27%) experienced a total of 11 different irAE categories (Table 2). Among them, grade 3 irAE occurred in 13 patients (16%). No patient experienced a grade 4 or higher AE. Of the grade 3 cases, 9 cases improved with nivolumab discontinuation and steroid treatment, whereas 3 cases with adrenal insufficiency required continuous hydrocortisone medication. In addition, 1 case with diabetes mellitus required permanent insulin administration. These 4 patients resumed nivolumab administration after the recovery from irAE.

**Table 2:** Summary of irAEs in metastatic renal cell carcinoma patients with nivolumab

	Grade 1	Grade 2	Grade 3	Grade 4 or 5
Interstitial pneumonia	1	3	1	
Rash	1	2	1	
Colitis			4	
Adrenal insufficiency			3	
Peripheral neuropathy		1	1	
Renal failure	1		1	
Hypothyroidism		1		
Fever			1	
Diabetes mellitus			1	
Hepatitis	1			
Pancreatitis		1		
Total	4 (5%)	8 (10%)	13 (16%)	0

### Correlation between the absolute eosinophil counts (AECs) value and irAE

**Fig. 1:**
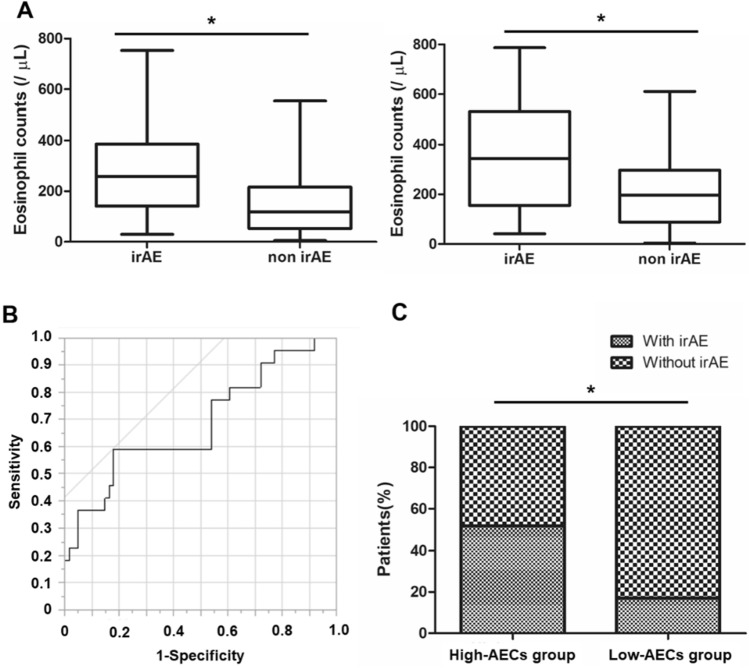
Comparison of absolute eosinophil counts between irAE group and non-irAE group. **A** The median absolute eosinophil counts (AECs) of patients with irAE at baseline and 4 weeks after the initiation of treatment were significantly higher than those without irAE (217 cells/uL versus 170 cells/uL, *p* < 0.001 and 357 cells/uL versus 211 cells/uL, *p* = 0.001). The median value is represented by the middle horizontal line in each box. The bottom and top of each box indicate the 25th and 75th percentiles, respectively. The ends of the whiskers indicate the minimum and maximum of all data. **B** Receiver operating characteristics curve analysis at 4 weeks after the treatment: area under curve = 0.686, sensitivity = 82.0%, specificity = 59.1%, cutoff value = 329 cells/µL. **C** When we divided the patients with the cutoff value (329 cells/µL) of counts, we observed that the incidence of irAE was significantly higher in high-AECs group (*n* = 12, 52%) compared with that in low-AECs group (*n* = 10, 17%) (*p* < 0.001)

**Fig. 2:**
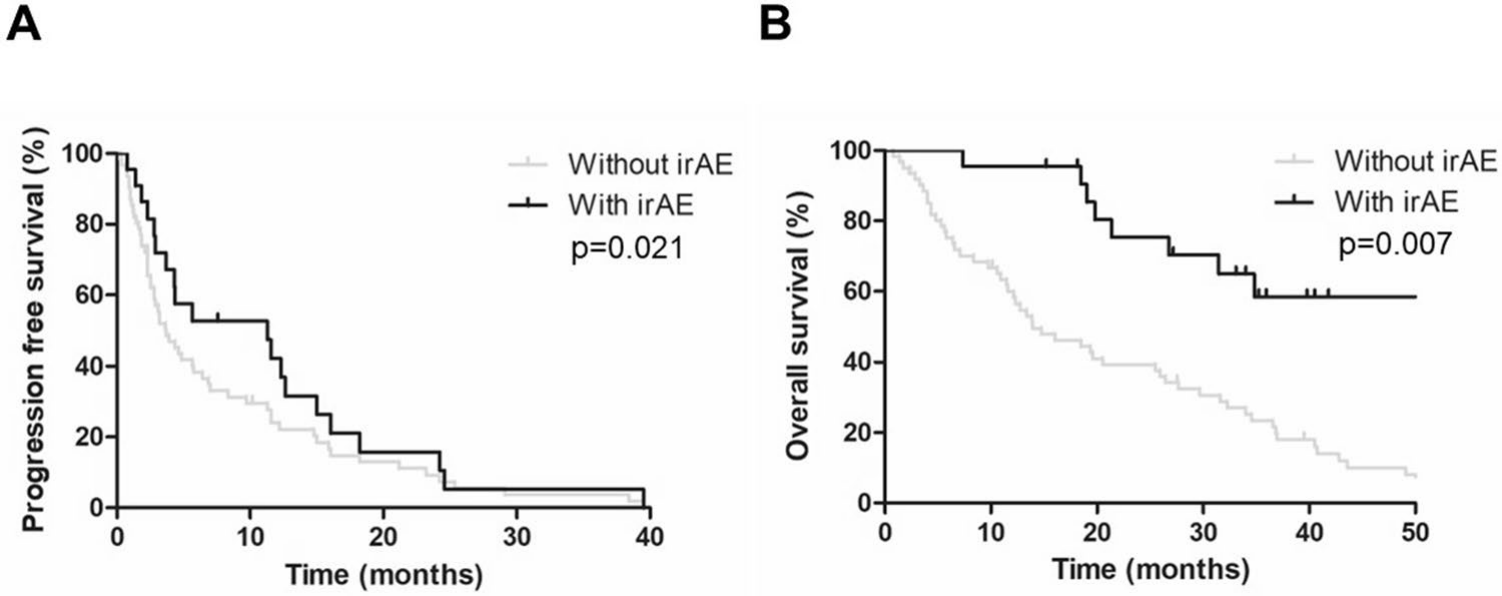
Correlation between the presence of irAEs and clinical outcomes. Kaplan–Meier survival curves show **A** progression-free survival (PFS) and **B** overall survival (OS) between irAE group and non-irAE group. Patients with irAE had significantly better PFS (median 10.9 months versus 3.7 months, *p* = 0.02) and OS (median NR versus 16.0 months, *p* = 0.007) than those without irAE

Next, we examined the whether the AECs value affected the grade of irAE. The median AECs value of the patients with irAE at baseline and 4 weeks after the initiation of treatment was significantly higher than those without irAE (217 cells/uL versus 170 cells/uL, *p* < 0.001 and 357 cells/uL versus 211 cells/uL, *p* = 0.001, Fig. 1A). The optimal cutoff value of AECs to differentiate the occurrence of irAE was 329 cells/µL at 4 weeks after the treatment, as determined by receiver operating characteristics (ROC) curve (Fig. 1B). The ROC curve was constructed based on the relationship between the occurrence of irAE and the AECs at week 4. According to this ROC curve, the AECs with the highest sensitivity and specificity were determined to be 329 cells/µL. When we divided the patients with the cutoff value (329 cells/µL) of AECs, we observed that the incidence of irAEs was significantly higher in high-AECs group (*n* = 12, 52%) compared with that in low-AECs group (*n* = 10, 17%) (*p* < 0.001, Fig. 1C). Moreover, when we examined the contribution of the irAE to improve the prognosis in mRCC patients, PFS and OS were significantly longer for patients who developed irAE than those who did not (*p* = 0.021 and *p* = 0.007, respectively, Fig. 2A and B).

### Correlation between the absolute eosinophil counts (AECs) value and clinical efficacy in patients with nivolumab

**Fig. 3:**
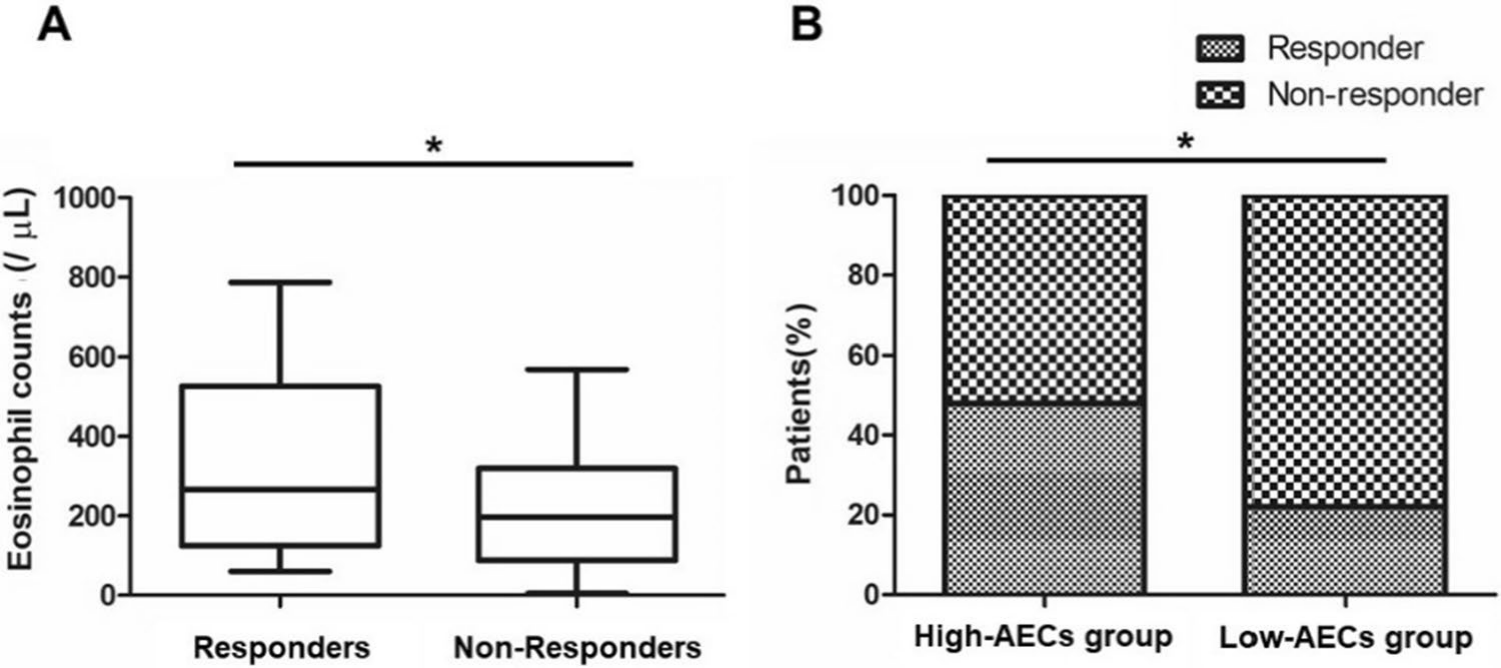
Comparison of absolute eosinophil counts between responders and non-responders. **A** Responders had significantly higher absolute eosinophil counts (AECs) at 4 weeks after the initiation of nivolumab compared to that in non-responders (*p* < 0.001). **B** When we divided the patients with the cutoff value (329 cells/µL) of AECs at 4 weeks after the initiation of nivolumab, the percentage of responder was significantly higher in high-AECs group (*n* = 11, 48%) compared with that in low-AECs group (*n* = 13, 22%) (*p* < 0.001)

**Fig. 4:**
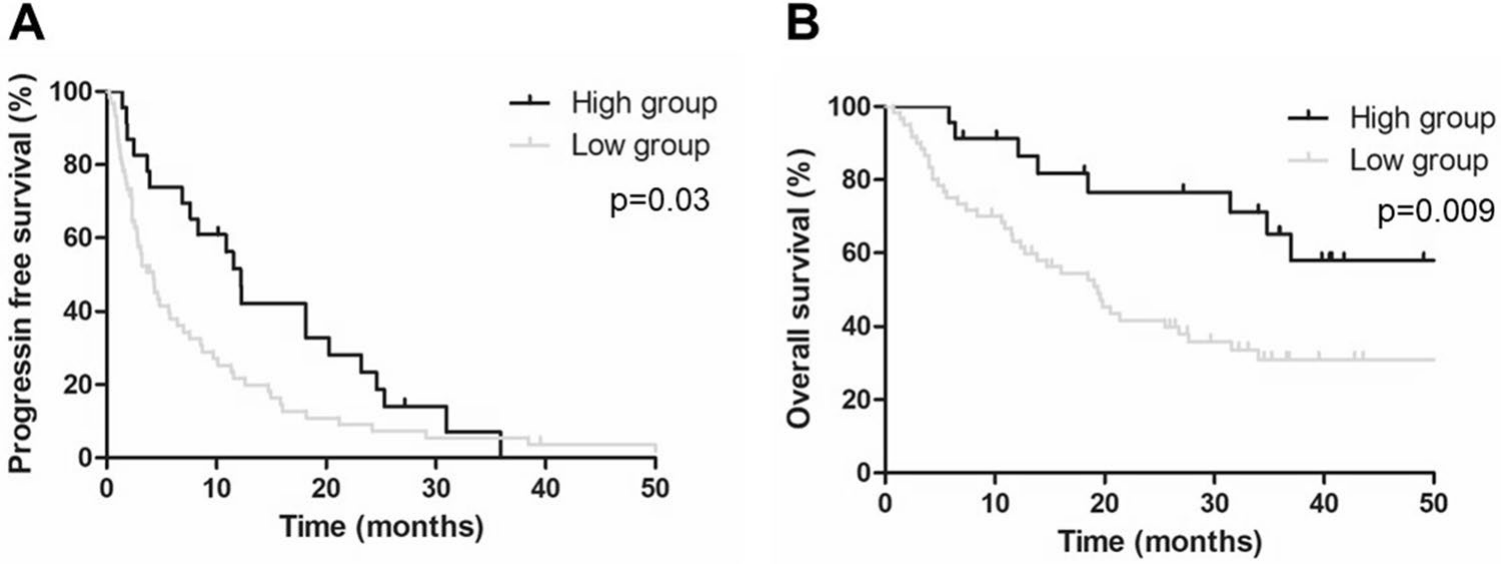
Correlation between absolute eosinophil counts at 4 weeks after the initiation of nivolumab and clinical outcomes. Kaplan–Meier survival curves show **A** progression-free survival (PFS) and **B** overall survival (OS) between high absolute eosinophil counts (AECs) group and low-AECs group. Patients with high-AECs had significantly better PFS (median 12.2 months versus 4.3 months, *p* = 0.03) and OS (median NR versus 16.4 months, *p* = 0.009) than those with low-AECs

We further investigated whether the AECs value affects the clinical efficacy of mRCC patients with nivolumab. As a result, responders exhibited a significantly higher AECs value than those of non-responders at 4 weeks after the treatment (242 cells/uL versus 178 cells/uL, *p* = 0.008, Fig. 3A). Importantly, when we divided the patients with the cutoff value (329 cells/µL), the percentage of responder was significantly higher in high-AECs group compared with that in low-AECs group (*p* < 0.001, Fig. 3B). The PFS and OS were significantly longer in patients with high-AECs value than those with low-AECs value (*p* = 0.03 and *p* = 0.009, respectively, Fig. 4).

Furthermore, the impacts of several clinicopathologic factors on PFS and OS in these 83 patients were evaluated (Table 3, 4). Univariate analysis identified AECs value, IMDC classification, the number of metastatic site and irAE were significantly associated with OS. Interestingly, in the multivariate analysis, higher AECs value was significantly associated with a better OS in patients (HR 0.401, 95% CI 0.176–0.909, *p* = 0.028).

**Table 3:** Univariate and multivariate logistic regression analysis for the prognostic factors of progression-free survival (*n* = 83)

	PFS			
Variable	Univariate analysis		Multivariate analysis	
	HR (95%CI)	*p* value	HR (95%CI)	*p* value
*Age*				
< 65	Reference			
≥ 65	1.028 (0.655–1.613)	0.90	–	–
*Gender*				
Male	Reference			
Female	1.487 (0.912–2.423)	0.11	–	–
*IMDC*				
≤ Intermediate	Reference			
poor	1.945 (1.141–3.315)	0.014	1.709 (0.892–3.271)	0.04
*Histology*				
Clear	Reference			
Non-clear	1.381 (0.781–2.444)	0.27	–	–
*Metastatic site*				
Single	Reference			
Multiple	1.575 (0.985–2.519)	0.058	–	–
*No. of treatment line*				
≥ 3rd	Reference			
2nd	0.684 (0.429–1.091)			
*irAEs*				
Absent	Reference			
Present	0.549 (0.322–0.921)	0.023	0.552 (0.246–1.239)	0.15
*4-week absolute eosinophil counts*				
< 329 cells/µL	Reference			
≥ 329 cells/µL	0.579 (0.348–0.965)	0.036	0.404 (0.188–0.865)	0.15

**Table 4:** Univariate and multivariate logistic regression analysis for the prognostic factors of overall survival (*n* = 83)

	OS			
Variable	Univariate analysis		Multivariate analysis	
	HR (95%CI)	*p* value	HR (95%CI)	*p* value
*Age*				
< 65	Reference			
≥ 65	0.822 (0.462–1.463)	0.51	–	–
*Gender*				
Male	Reference			
Female	1.498 (0.825–2.719)	0.18	–	–
*IMDC*				
≤ Intermediate	Reference			
poor	1.977 (1.055–3.704)	0.033	1.554 (0.813–2.970)	0.18
*Histology*				
Clear	Reference			
Non-clear	1.685 (0.873–3.252)	0.12	–	–
*Metastatic site*				
Single	Reference			
Multiple	2.103 (1.176–3.759)	0.012	2.072 (1.121–3.829)	0.020
*No. of treatment line*				
≥ 3rd	Reference			
2nd	0.892 (0.460–1.607)	0.70	–	–
*irAEs*				
Absent	Reference			
Present	0.365 (0.170–0.783)	0.010	0.567 (0.251–1.280)	0.17
*4-week absolute eosinophil counts*				
< 329 cells/µL	Reference			
≥ 329 cells/µL	0.376 (0.174–0.808)	0.012	0.400 (0.176–0.909)	0.029

## Discussion

The landscape of oncology has been drastically revolutionized by the emergence of ICIs, significantly enhancing the prognosis of previously incurable cancers [17]. However, the response rate to ICIs for mRCC is still limited [18–22], and treatment-related irAE often causes discontinuation of ICIs. Therefore, it is critical to identify biomarkers to predict the response of patients to ICIs to enable a precision medicine approach. Although the association between peripheral eosinophil counts and the response to ICIs has been observed in several types of cancer, the evidence is still limited due to the variety of evaluation methods of eosinophils. Hence, in this study, we performed the data analysis of mRCC patients treated with nivolumab and clarified some evidences about AECs affecting adverse events and clinical outcomes.

First, we found mRCC patients with irAE had higher AECs value compared to those without irAE at baseline and 4 weeks after treatment (Fig. 1A). Our findings partially align with the data published by Ma et al. reported that irAE was more likely to occur in patients with higher AECs at the start of treatment in multiple cancer types treated with PD-1 or PD-L1 inhibitors [15]. Giommoni et al. also reported that high-AECs at the start of treatment were a significant risk factor for the occurrence of irAE in 168 cancer patients including 43 RCC patients [16]. In the present study, we first confirmed continuous activation of eosinophils in patients with irAE throughout nivolumab treatment, suggesting that it may be useful to prepare for the occurrence of irAE by regularly monitoring AECs.

Secondly, our results demonstrated superior efficacy outcomes in patients with high-AECs value at 4 weeks after treatment, as evidenced by improved PFS and OS in this group (Fig. 3, 4). In this decade, several studies reported the relationship between eosinophils and the efficacy of ICI treatment in several types of cancer with a focus on the ratio of pre- and post-treatment eosinophils [9–13]. In our study, we first propose that absolute value of eosinophils counts at 4 weeks after the treatment clearly allows identifying early surrogate biomarkers in the peripheral blood for predicting clinical responses to anti-PD-1 therapy. Eosinophils infiltrate multiple tumors and regulate tumor progression either directly by interacting with tumor cells or indirectly by shaping the tumor microenvironment [23]. Carretero et al. reported that eosinophils evoke further immune response with CD8 + T cells in tumor microenvironment by producing C–C motif chemokine ligand 5 (CCL5), C–X–C motif chemokine ligand 9 (CXCL9), and CXCL10 [24]. These results may support the potential mechanism of anti-cancer immune response of peripheral eosinophils in cancer patients.

There are several limitations to this study. First, our small sample size may limit the generalization for our findings to other types of cancer. Second, we only enrolled mRCC patients with anti-PD-1 therapy. Given that ICI combination therapies have become standard first-line treatments in mRCC field, further prospective studies are required to consolidate our results.

## Conclusion

We found that the high absolute eosinophil counts at 4 weeks after the start of nivolumab were a prominent prognostic marker associated with clinical outcome in mRCC patients.

## Supplementary Information

Below is the link to the electronic supplementary material.

Kaplan–Meier survival curves show (A) progression-free survival and (B) overall survival of all patients. (PDF 145 KB)
