# Supplementary figures and images for: The prognostic impact of peripheral blood eosinophil counts in metastatic renal cell carcinoma patients treated with nivolumab
Source: Clin Exp Med. 2024 May 23;24(1):111. doi: 10.1007/s10238-024-01370-8 (PMC11116198; doi:10.1007/s10238-024-01370-8)

Supplementary Fig.1

A

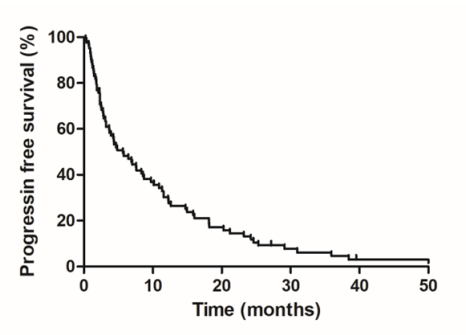

B

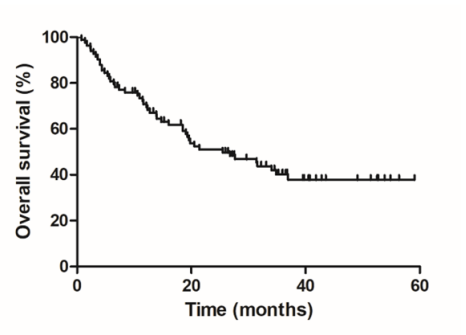

Supplement: Supplementary file 1 — Kaplan–Meier survival curves show (A) progression-free survival and (B) overall survival of all patients. (PDF 145 KB) [file 10238_2024_1370_MOESM1_ESM.pdf]
